# Study on the Effect of Laser Power on the Microstructure and Properties of Cladding Stellite 12 Coatings on H13 Steel

**DOI:** 10.3390/ma17246098

**Published:** 2024-12-13

**Authors:** Qianjie Wang, Xuedao Shu, Haijie Xu, Sheng Xu, Song Zhang

**Affiliations:** 1 Faculty of Mechanical Engineering & Mechanics, Ningbo University, Ningbo 315221, China; dagouzi0302@163.com (Q.W.); xuhaijie@nbu.edu.cn (H.X.); 2 School of Information and Intelligent Engineering, Ningbo City College of Vocational Technology, Ningbo 315221, China; xusean@126.com; 3 College of Mechanical and Automotive Engineering, Ningbo University of Technology, Ningbo 315221, China; zhangsong@nbut.edu.cn

**Keywords:** laser cladding, Stellite 12 alloy, H13 mold steel, microstructure, wear resistance

## Abstract

To address the issue of cracking in aluminum extrusion dies during operation, this study employs laser cladding technology to modify the surface of these dies. This modification aims to enhance their hardness and friction resistance. Laser cladding technology was utilized to coat the surface of H13 steel with Stellite 12, a cobalt-based alloy, at varying laser power levels. The surface formation quality, microstructural organization, phase composition, microhardness, and wear resistance of the coatings were investigated using optical microscopy, scanning electron microscopy, energy-dispersive spectroscopy, X-ray diffraction (XRD), microhardness testing, and confocal microscopy. The results indicated that as the laser power increased, the surface formation quality of the coating gradually improved, while the dilution rate of the coating increased. Changes in the phase composition and microstructure were not significant, and both microhardness and wear resistance initially increased before decreasing. Optimal process parameters for achieving good surface formation quality, high microhardness, and strong wear resistance were found to be a laser output power of 2200 W, scanning speed of 10 mm/s, feeding rate of 1.2 r/min, and overlap rate of 40%. The results indicate that the coating applied to the surface of H13 steel using Stellite 12 enhances the performance of aluminum extrusion dies.

## 1. Introduction

H13 mold steel is a chromium–molybdenum hot work tool steel that is widely used in high-temperature environments such as forging, extrusion, stamping, and casting. This material is preferred for its excellent resistance to thermal fatigue, erosion, and wear [1,2,3,4]. To extend its service life and prevent premature failure under harsh conditions, the surface modification of specific areas of H13 steel is essential. Among the various surface modification techniques, recent years have seen increasing interest in the use of multiple materials, including ceramic composites, via methods such as laser remelting, alloying, or cladding [5,6,7,8].

Compared to traditional methods, laser cladding technology offers several significant advantages: it produces a smaller heat-affected zone, involves low heat input, effectively reduces part distortion, and allows for faster processing speeds with greater precision [9]. Particularly, laser cladding technology is attracting increasing attention in the industry due to its low heat input [10,11,12,13], low dilution rate, high microstructural density [14,15,16,17], strong bonding between the coating and substrate, and the flexibility to adjust the coating’s composition, thickness, and properties.

Chen et al. [18] prepared H13 steel composites reinforced with coarse TiC particles (greater than 50 μm) at various ceramic volume fractions on the surface of H13 steel. They investigated the effect of ceramic volume fraction on the microstructure and hardness of the TiC/H13 composites and found that combining preheating with a reduced laser beam scanning speed effectively reduced crack formation when producing composites with high TiC volume fractions. Kattire et al. [19] cladded CPM 9 V steel powder onto the surface of H13 steel and discovered that vanadium carbide particles were embedded in martensite and retained austenite, resulting in an average hardness of the cladding layer that was four times that of the substrate, while also inhibiting crack propagation, thereby extending mold life. Liu et al. [20] studied the microstructure and mechanical properties of Stellite 6 coatings at high power and found that, under high laser power and high powder feed rates, there were fewer internal thermal cracks in the cladding layer. This process reduced the solidification and cooling rates of the melt pool, leading to the phase transformation of γ-Co to ε-Co. Consequently, the dendritic regions were primarily composed of γ-Co, while the interdendritic regions contained small amounts of ε-Co and Cr_23_C_6_. Wang et al. [21] prepared Stellite 6 coatings and examined the macro-morphology and mechanical properties of the coatings, initially researching the process parameters for single-pass laser cladding. They determined that optimal coating performance was achieved with a laser power of 1400 W and a scanning speed of 4 mm/s while also exploring the effects of different spot types on coating performance. Gorka et al. [22] prepared a Co-Cr-W-C-Ti alloy (Stellite 6 type) cladding layer using an in situ synthesis method. Their study found that the optimal concentrations of Ti and C in the Co-Cr-W-C alloy facilitated the formation of titanium carbide, significantly enhancing erosion resistance at low impact angles. The concentrations of titanium, carbon, and tungsten in the molten metal pool can influence the morphology and size of the reinforcing phase in the form of composite carbide (Ti, W)C, thereby affecting the alloy structure.

Currently, Stellite 12 series alloys are widely utilized; however, their application on the surfaces of aluminum extrusion molds remains relatively limited. This study primarily focuses on the surface reinforcement of the working surfaces of aluminum extrusion molds. The objective of this paper is to create cladding layers on the surface of H13 steel molds using varying laser powers, thereby investigating how the microstructure and properties of the cladding layers change with different laser powers. This research aims to identify suitable laser power parameters that can yield cladding layers with optimal surface morphology, refined microstructure, high hardness, and strong wear resistance, thereby providing technical support for the laser cladding of extrusion mold surfaces. Compared to other, similar studies, the selected material, Stellite 12, is more innovative and unique. Additionally, the application in the aluminum extrusion mold field is more detailed and precise, making the results both universal and practical, and we utilize the results of the experiment to modify the surface of the extrusion mold, extend the mold’s service life, and contribute to reducing the enterprise’s costs.

## 2. Experimental Section

### 2.1. Materials and Specimens

**Figure 1:**
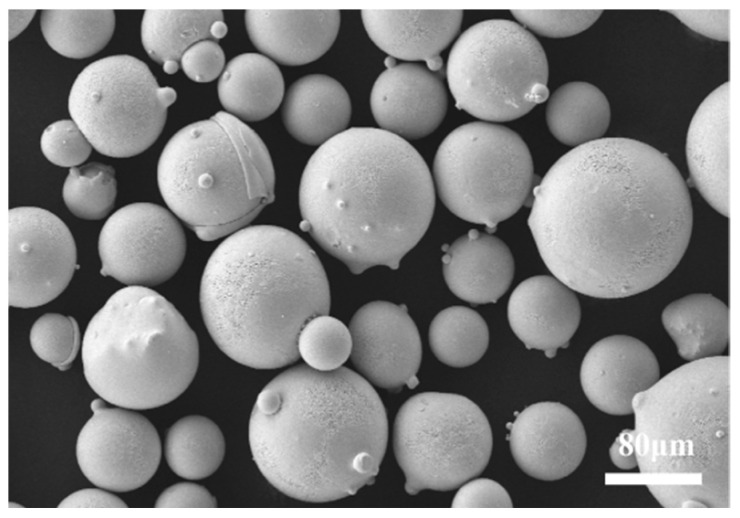
SEM image of Stellite 12 powder.

**Figure 2:**
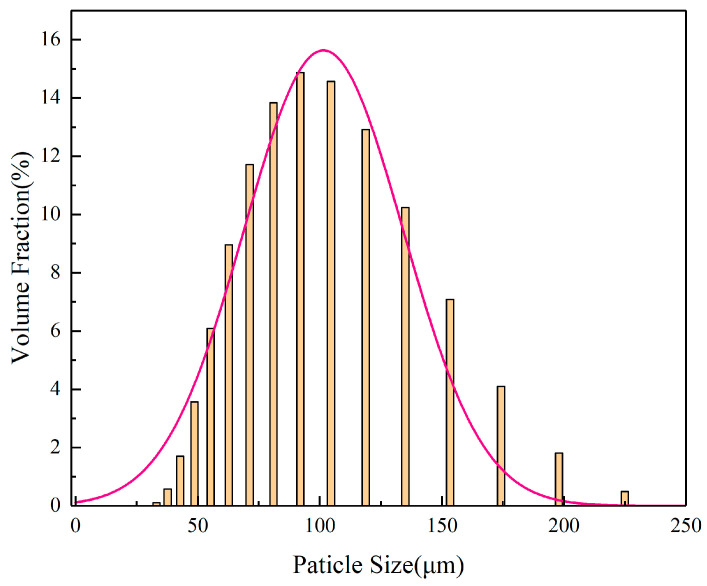
Particle size distribution of Stellite 12 powder.

A Stellite 12 Co-based alloy coating was prepared on H13 steel (100 × 100 × 10 mm) using laser cladding technology. Before cladding, the surface of the substrate was polished to remove the oxidation layer, followed by sanding with 200- and 400-grit sandpaper to achieve a smooth finish. Finally, ethanol was used for cleaning, and the substrate was air-dried for later use. The cladding material chosen was spherical Stellite 12 alloy powder with an average particle size ranging from 100 to 325 mesh [15]. The scanning electron microscopy (SEM) image of Stellite 12 powder particles had a spherical shape with few satellite particles attached (as shown in Figure 1). The particle size distribution of the powder was tested using the Mastersizer 3000 laser particle sizer from Malvern Panalytical Limited (Great Malvern, UK). The composition of the Stellite 12 powder is detailed in Table 1. It is obvious that the particle size distribution of the powder conforms to the standard normal distribution (as shown in Figure 2). Prior to cladding, the powder needs to be dried in a vacuum oven at 100 °C for 2–4 h to remove any internal crystallization water [23].

**Figure 3:**
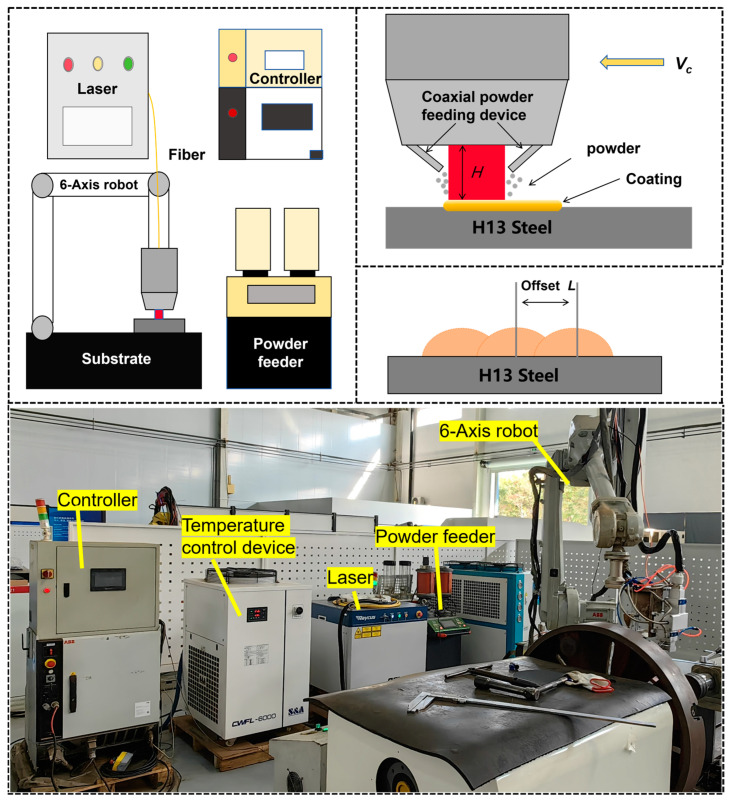
Schematic diagram of cladding equipment and process.

The laser cladding equipment used in this experiment was an integrated system (as shown in Figure 3) from Ningbo Haitian Laser Technology Co., Ltd., Ningbo, China. It includes a 6 kW laser (with a power range of 0–6000 W) paired with a six-axis robotic arm from ABB to drive the laser cladding head (which is designed with a square spot shape of 3 × 3 mm). This setup allows for spatial displacement conversion, enabling synchronous powder feeding during the cladding experiments on the substrate. Following extensive preliminary trials, it was determined that a scanning speed of 10 mm/s, combined with a powder feeding rate of 1.2 r/min and a laser power range of 1800 W to 2600 W, yields a cladding layer with good surface quality. The specific parameters for the laser cladding process are detailed in Table 2. An overlap rate of 0.4 was selected as the process parameter for this experiment, diverging from the traditional laser cladding process that typically utilizes an overlap rate of 0.5. This decision was based on extensive testing conducted in our preliminary stages, which showed its superior performance in significantly reducing crack formation.

### 2.2. Characterization

The samples obtained from laser cladding were cut into 10 mm × 10 mm × 10 mm cubes using a wire cutting machine. Next, the cross-section of the cladding layer was polished sequentially with sandpaper of grit sizes 400, 600, 800, 1000, 1200, 1500, and 2000, concluding with the use of a 3 μm abrasive agent to achieve a mirror-like finish (Ra0.02–0.16). After polishing, the samples were examined under an optical microscope (OM) to identify any internal defects. Subsequently, the samples underwent chemical etching using an etching solution composed of 2 g of CuSO_4_, 10 mL of HCl, and 10 mL of H_2_O for 10 s [24,25,26,27]. After etching, the samples underwent ultrasonic cleaning, followed by cleaning with anhydrous ethanol and drying with a blower.

The microstructural analyses of the hardfacings were performed on a Zeiss (G360) scanning electron microscope (SEM) (Carl Zeiss AG, Oberkochen, Germany) with an energy-dispersive spectrometer (EDS). The hardness of the coating was measured using an HYHV-1000AT Vickers hardness tester (kason, Qingdao, China), with a load of 300 g and a loading time of 15 s, progressing from the surface of the coating towards the substrate. The hardness test results were measured three times in total and the average value was calculated. Subsequently, the samples were again cut into 10 mm × 10 mm × 10 mm blocks using a wire cutting machine. The surface of the cladding layer was ground down by 0.3 mm using a grinding machine, followed by sequential polishing with sandpaper of grit sizes 400, 600, 800, 1000, 1200, 1500, and 2000, concluding with the use of a 3 μm abrasive agent to achieve a mirror-like finish (Ra0.02–0.16). Phase identification of the coating was performed using a Bruker (D8 ADVANCE) X-ray diffractometer (XRD) (Bruker, Woltzbach, Germany), with the scanning angle set from 10° to 90° and a step size of 2°·min^−1^ (tube voltage of 40 kV, tube current of 30 mA, Cu target).

Finally, friction and wear tests were conducted using a CMS-01B diesel lubrication high-frequency reciprocating test machine (Beijing Chaoyang Gaoke Application Technology Institute Co., Ltd. Beijing, China), equipped with a 6 mm diameter Si_3_N_4_ friction ball (92HRC, Ra0.02 μm). Subsequently, the samples were again cut into 10 mm × 10 mm × 10 mm blocks using a wire cutting machine. The surface of the cladding layer was ground down by 0.3 mm using a grinding machine, followed by sequential polishing with sandpaper of grit sizes 400, 600, 800, 1000, 1200, 1500, and 2000, concluding with the use of a 3 μm abrasive agent to achieve a mirror-like finish (Ra0.02–0.16). The machine operated at a frequency of 50 Hz, a sliding distance of 1 mm, a load of 100 g, and a wear duration of 60 min, and no oil was used for lubrication. The entire experiment was conducted at room temperature. The Si_3_N_4_ ball showed minimal changes before and after the friction and wear experiment. Additionally, to accurately control the experimental variables, we flipped the Si_3_N_4_ ball at a specific angle before completing each trial and used an untested surface for the friction experiments to ensure the rigor of this study. Through these steps, a comprehensive analysis of the microstructure, composition, and properties of the laser cladding layer was performed. The laser scanning confocal microscope (LSM 900, Carl Zeiss AG, Oberkochen, Germany) was used to measure the size of the wear scar and the wear volume. The wear scar morphology and sizes can be obtained using the wear morphology scanning function of the LSM 900. Subsequently, the wear volume can be determined by the volume measurement function. The volume measurement was conducted three times in total, and the average of these measurements was calculated.

## 3. Results and Discussion

### 3.1. Macroscopic Morphology of Fused Cladding Layer

**Figure 4:**
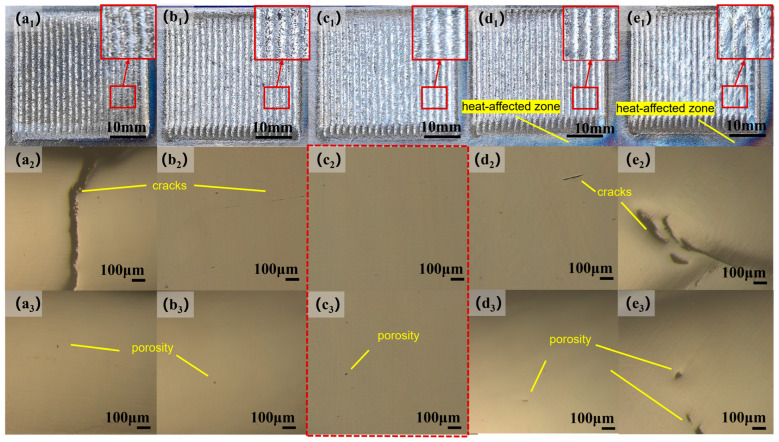
(**a_1_**–**e_1_**) Surface morphology images of the cladded layer and its local area magnification; (**a_2_**–**e_2_**,**a_3_**–**e_3_**) morphological images of the cladded layer under optical microscopy (OM), using the same magnification to show the internal defects of the cladding layer, and the size and shape of the cracks and porosity can be obviously compared.

As shown in the figure, Figure 4(a_1_–e_1_) display the Stellite 12 cladding layers fabricated at different laser powers (1800 W, 2000 W, 2200 W, 2400 W, and 2600 W). All cladding layers exhibit a metallic luster and have no significant surface defects. Additionally, the surface quality of the coating improves gradually with increasing laser power, because at lower laser powers (1800 W and 2000 W), some incompletely melted particles are observed on the surface of the cladding layer (as shown in Figure 4(a_1_–e_1_)). This phenomenon arises from the insufficient energy input from the low-power laser, which does not fully melt all the powder, resulting in residual particles adhering to the surface. In contrast, at higher laser powers (2400 W and 2600 W), the surface quality of the cladding layer is better, with no noticeable particles present; however, the heat-affected zone (HAZ) expands more significantly. This is attributed to the increased energy input from the high-power laser, particularly evident for carbon steels such as H13 steel, where the heat-affected zone is more pronounced (see Figure 4(d_1_,e_1_)). At a laser power of 2200 W, the surface of the cladding layer is well formed, and no significant cracks are observed internally.

### 3.2. Defect Analysis of Fused Cladding Layer

To further analyze the microscopic defects within the coating, we performed a standard polishing procedure and used an optical metallographic microscope (OM) to observe the samples of the Stellite 12 cladding (Figure 4). Figure 4(a_2_–e_2_) present the OM images of the coating area. At a low laser power of 1800 W, as shown in Figure 4(a_2_), a crack can be clearly observed extending from the bonding area to the surface of the coating. This occurs due to the low input power of the laser leading to an excessively low dilution rate, which results in a weak bond between the coating and the substrate, thereby forming cracks. Porosity is another common defect, as illustrated in Figure 4(a_3_–e_3_). As the laser power increases from 1800 W to 2600 W, the size of the pores tends to increase. The higher laser power enhances the temperature gradient between the cladding layer and the substrate, causing more gasses to become trapped and unable to escape the coating in time, thus forming larger pores. Further analysis shows that when the laser power increases from 1800 W to 2200 W, the length and width of the internal cracks in the coating decrease, reducing the number of pores (as shown in Figure 4(a_2_–c_2_)). However, when the laser power is increased from 2200 W to 2600 W, the length and width of the cracks again increase, and the internal pores also enlarge (as shown in Figure 4(c_2_–e_2_)). This phenomenon is attributed to the accumulation of pore defects caused by high laser power, which leads to the formation of cracks at the bottom. In summary, as the laser power changes from 1800 W to 2600 W, the internal crack defects initially decrease and then increase, with the fewest cracks and pores observed at 2200 W. The location of the cracks also shifts from the top of the coating to the bottom near the bonding area.

### 3.3. Microstructural Analysis of the Cladding Layer

**Figure 5:**
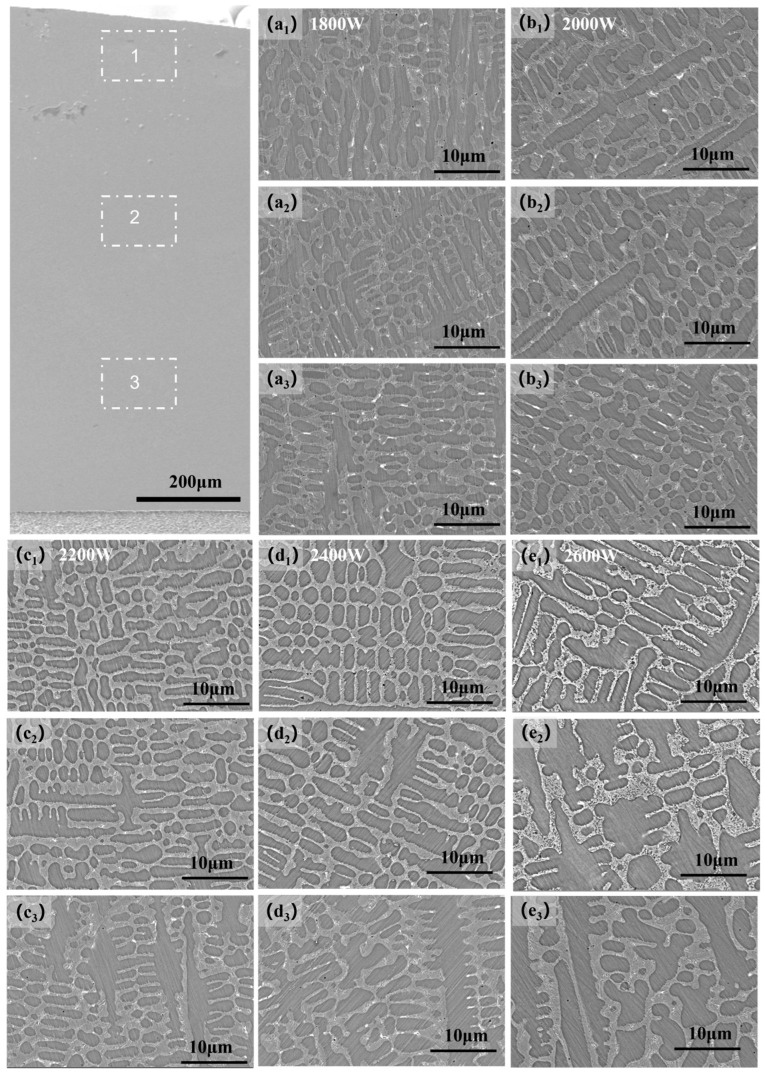
SEM images of the microstructure of the cladded layer at the top, middle, and bottom under different power settings. In the figure, 1, 2, and 3 correspond to the top, middle, and bottom layers of the coating, respectively. Panels (**a_1_**–**e_1_**) present the scanning electron microscopy (SEM) images of the top layer, while panels (**a_2_**–**e_2_**) display the SEM images of the middle layer, and panels (**a_3_**–**e_3_**) depict the SEM images of the bottom layer.

Figure 5 shows the morphological characteristics of the cladding layer cross-section at the top, middle, and bottom at the same location under different laser power levels. At lower laser powers (1800–2000 W), the input energy into the molten pool is low, resulting in many irregular grains that have not fully developed. As the laser power increases to 2400–2600 W, the irregular grains at the top of the coating transform completely into regular columnar and cellular grains. At a power of 2200 W, the grain arrangement in the coating is dense and well formed. Overall, as the laser power gradually increases, there is a noticeable trend of grain growth, which may be attributed to the accumulation of heat during the high-power processing leading to coarser grain sizes.

The microstructure of the coating gradually evolves from the bottom to the top, presenting planar crystals, cellular crystals, columnar crystals, dendrites, and equiaxed crystals in succession. These changes are primarily influenced by the ratio of the temperature gradient to the solidification rate (G/R ratio). In the laser cladding process, rapid heating and cooling cycles create a temperature gradient (G) within the cladding layer. The powder solidifies quickly during this cooling solidification process; however, the cooling and solidification rates (R) inside and outside the coating differ, as characterized by the G/R ratio. A higher G/R ratio is conducive to the growth of planar crystals along the interface direction, whereas a lower G/R ratio favors the formation of columnar and cellular crystals perpendicular to the interface direction. This phenomenon reflects the directional heat flow between the coating and the substrate during the solidification process of the molten pool. In different regions (bottom, middle, and top), the crystal morphology changes with variations in the G/R ratio. The high G/R ratio at the bottom facilitates the generation of planar crystals, whereas a reduced ratio leads to a decrease in cellular crystals and the formation of dendrites. The lowest G/R ratio in the top region promotes the development of equiaxed crystals and dendrites, which typically appear toward the end of the solidification process [11,28,29,30].

As shown in Figure 5a_3_–e_3_, the high G/R ratio in the bottom region promotes the growth of planar crystals along the interface, while a decrease in the G/R ratio leads to the formation of columnar and cellular crystals that are nearly perpendicular to the interface. This phenomenon arises from the directional heat flow between the coating and the substrate during the solidification and crystallization processes of the molten pool. A further reduction in the G/R ratio, as illustrated in Figure 5a_2_–e_2_, results in a decrease in the number of cellular crystals at the bottom, accompanied by the emergence of dendrites. The undercooling effect induced by solute concentration facilitates the formation of these dendrites. In Figure 5a_1_–e_1_, when equiaxed crystals begin to grow on the dendrites, the G/R ratio in the top region reaches its lowest point.

### 3.4. Phase Analysis of Cladding Layer

**Figure 6:**
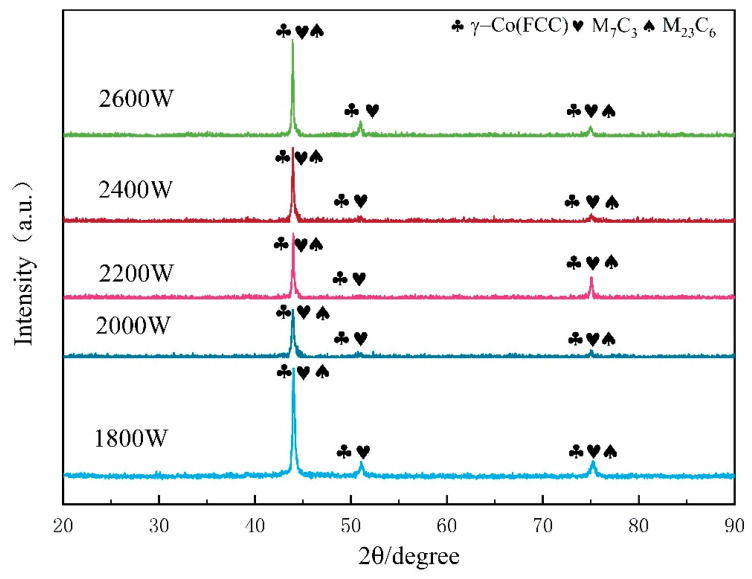
XRD patterns of the cladded layer.

According to the X-ray diffraction (XRD) analysis results (as shown in Figure 6), the cladding layer under different laser power conditions mainly consists of γ-Co (face-centered cubic structure), M_7_C_3_, and M_23_C_6_ (where M represents Co, Cr, Mo, and W). The presence of these phases is not significantly correlated with the variation in laser power, indicating that laser power has little effect on the phase composition of the cladding layer.

During the cladding process, elements such as W, Cr, and Ni from the Stellite12 powder dissolve into the cobalt matrix, increasing the lattice constant of cobalt. This leads to a leftward shift in the position of the Co peak observed in the experiments, reflecting the effects of solid solution strengthening. Notably, cobalt undergoes an allotrope transformation at different temperatures: above 360 °C, cobalt adopts a face-centered cubic (FCC) structure, referred to as γ-Co; below 360 °C, it adopts a hexagonal close-packed (HCP) structure, referred to as ε-Co. In the rapid laser cladding process, the fast laser scanning speed and minimal heat accumulation result in quick heat dissipation from the substrate and rapid solidification of the molten pool. Additionally, the presence of Ni is crucial for stabilizing the face-centered cubic structure. The Stellite 12 alloy contains approximately 2.21% Ni, which limits the transformation of γ-Co to ε-Co in the cladding layer. This allows a significant amount of γ-Co to remain stable at high temperatures and to persist in the solidified coating at room temperature, thereby explaining the predominance of γ-Co observed in the XRD patterns. Moreover, under the influence of the high-energy laser beam, carbon elements that precipitate combine with other alloying elements (Co, Cr, Mo, W) to form various carbides, which are distributed between dendrites. This contributes to dispersion strengthening, significantly enhancing the mechanical properties of the cladding layer [31,32,33,34].

**Figure 7:**
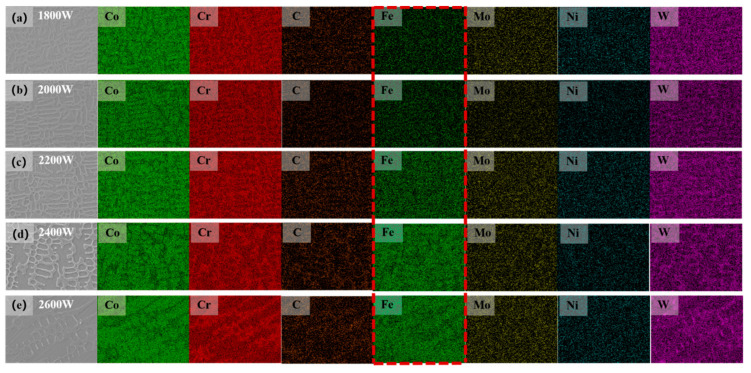
EDS surface mapping images of the cladded layer. Figures (**a**–**e**) show coatings prepared at 1800 W, 2000 W, 2200 W, 2400 W, and 2600 W. The dotted square exhibit significant variations in the concentration of iron (Fe) elements.

Based on the X-ray diffraction (XRD) analysis, a detailed elemental distribution analysis of the coating was conducted through energy-dispersive spectroscopy (EDS) scanning, focusing on the differences in elemental distribution across various regions. Figure 7a–e illustrate the elemental distribution in the central region as the laser power ranges from 1800 W to 2600 W. The analysis reveals that Fe and Co elements are enriched in the dendritic region, indicating that Co, as the primary phase, precipitates first during the rapid non-equilibrium solidification process, followed by the accumulation of alloying elements such as Fe within the dendrites. In contrast, elements such as C, Cr, and W tend to accumulate in the interdendritic regions, likely related to their segregation behavior during the solidification process. As the laser power increases, the brightness of the Fe element gradually intensifies. This phenomenon is primarily due to the increased energy input into the molten pool with higher laser power, which extends the duration of the molten pool. Consequently, this results in the greater diffusion of Fe from the substrate into the cladding layer, leading to a significant increase in the brightness of the Fe element with rising laser power. This observation further supports the idea that the diffusion and accumulation behaviors of different elements during the cladding process are closely related to laser power. In summary, this study not only reveals how different elements affect their distribution characteristics during the cladding process but also provides important experimental evidence for optimizing the laser cladding process. By adjusting the laser power, it is possible to effectively control the composition and microstructure of the coating, thereby enhancing the material’s performance and applicability.

According to the results of X-ray diffraction (XRD) and energy-dispersive spectroscopy (EDS), we conclude that carbides such as M_7_C_3_ and M_23_C_6_ are generated in the cladding layer, with most of the generated carbides distributed in the middle and bottom sections. Based on this theory, we speculate that carbide formation and diffusion in the cladding layer enhance its hardness, with the hardness improvement in the middle and bottom regions being greater than that in the top.

### 3.5. Coating Microhardness Analysis

**Figure 8:**
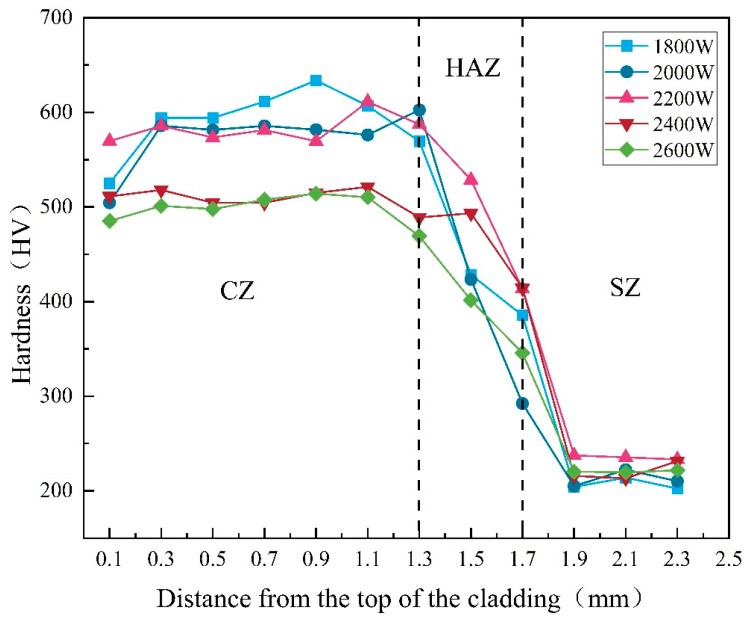
Microhardness distribution map of the cladded layer.

According to the data presented in the charts, the influence of different laser powers on the Stellite 12 coating indicates that, with increasing laser power, the overall hardness of the coating decreases (as shown in Figure 8). Additionally, changes in the internal structure and chemical composition of the coating significantly affect the distribution of hardness. The average microhardness of the substrate is 224.32 HV. When the laser power is set at 1800 W, the average microhardness of the cladding layer reaches 590.72 HV, the highest among the five groups of laser powers. However, when the laser power is increased to 2600 W, the microhardness drops to 487.54 HV, the lowest value observed.

Across all laser powers, the trend of coating hardness varies from the surface to the substrate, initially increasing and then decreasing. This phenomenon can be attributed to several combined factors: first, during the coating process, some gasses are not completely expelled, leading to defects such as porosity during solidification, which reduces hardness. Secondly, the increase in laser power decreases heat loss within the coating, allowing more time for grain growth, which enlarges the grain size and subsequently lowers the hardness of the cladding layer.

Furthermore, at lower laser powers, the presence of fine grains within the coating helps enhance hardness. In the mid and bottom regions, the presence of strengthening elements such as Cr, Ni, and W contributes to localized improvements in hardness due to their favorable distribution within the coating. The results of the hardness test confirm the findings of the phase analysis, showing that the hardness of the middle and bottom sections of the cladding layer is significantly improved and is higher than that of the top. This enhancement is attributed to the dispersion and strengthening effects associated with the formation of M_7_C_3_ and M_23_C_6_.

### 3.6. Analysis of Coating Friction and Wear

**Figure 9:**
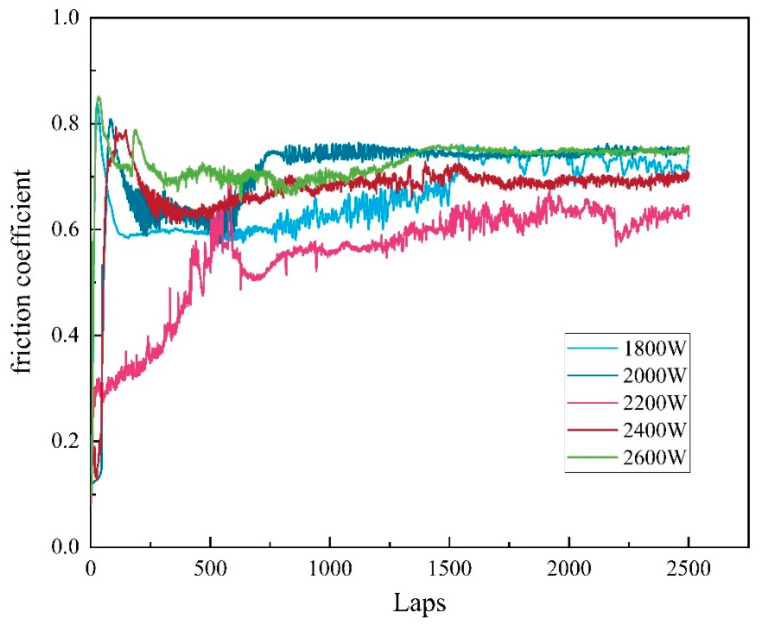
Friction coefficient profile of the cladded layer.

According to the operational requirements of extrusion molds, the wear resistance of the molds must meet strict standards. After conducting friction and wear experiments, the surface morphology of the samples was observed using a confocal microscope. The analysis of the friction and wear experiments, along with the friction coefficient curves, concludes that the trends of friction coefficients over time are similar at different laser power levels (as shown in Figure 9) [35,36].

**Figure 10:**
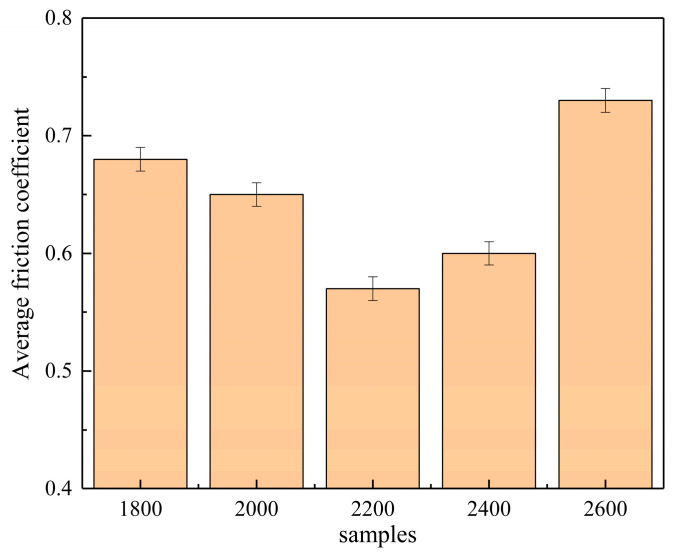
Average friction coefficients.

**Figure 11:**
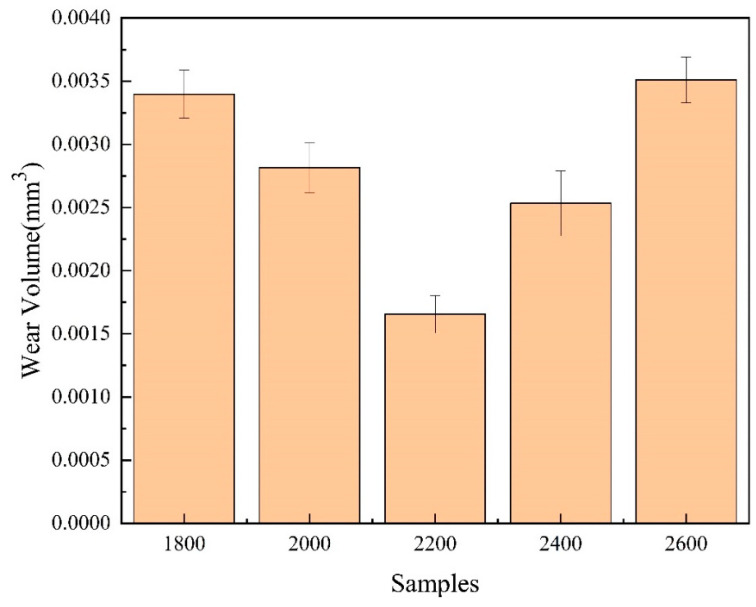
Wear volume of the cladded layer.

In the initial stage of the experiments, the presence of small surface protrusions resulted in a smaller contact area and stress concentration, causing significant fluctuations in the friction coefficient. As the friction time increased, these protrusions gradually wore down, making the contact surface smoother. Eventually, the friction coefficient stabilized, entering a steady-state friction phase. The friction coefficient can indirectly reflect the roughness and wear resistance of the overlay layer. Generally, a lower friction coefficient indicates a smoother surface of the overlay layer, which correlates with better wear performance. According to the data, the friction coefficients of the coatings at different laser power levels were 0.68, 0.65, 0.57, 0.60, and 0.73 (as shown in Figure 10). Under these conditions, the coating prepared at 2200 W exhibited the lowest friction coefficient, demonstrating superior wear resistance and smoothness. Therefore, measuring the friction coefficient provides important insights for evaluating the performance of overlay layers under the working conditions of extrusion molds at different laser power levels, aiding in optimizing process parameters and improving the overlay process. According to the data presented in Figure 10, the wear volume exhibits a trend of initially decreasing and then increasing with varying laser power. Specifically, at 2600 W, the wear of the overlay layer reaches its maximum value, while the friction loss at 2200 W is at its minimum, recorded at only 0.0162 mm^3^ (as shown in Figure 11). This variation is primarily attributed to the differences in the internal structure of the coatings [37,38,39,40].

The coating produced at a laser power of 2200 W features a dense internal structure with well-defined grains, contributing to enhanced hardness and wear resistance. As a result, it demonstrates the lowest friction loss during the friction tests. In contrast, coatings produced at lower power levels (1800 W to 2000 W) exhibit incomplete grain growth, leading to a decline in friction performance. At higher power levels (2400 W to 2600 W), the larger grain size may reduce hardness and wear resistance, resulting in increased friction loss. Therefore, by adjusting the laser power to control the internal microstructure of the overlay layer, it is possible to effectively optimize the friction performance of the coating, thereby improving its wear resistance and long-term stability in practical applications.

**Figure 12:**
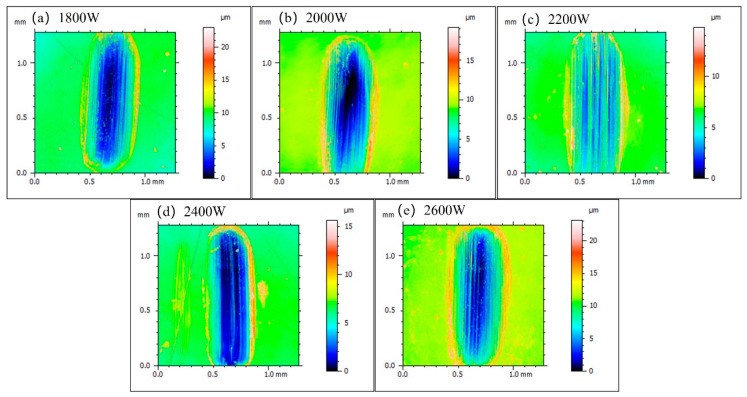
Morphology of the wear scars. Figures (**a**–**e**) show the wear scars morphology under different laser power.

Figure 12 shows the wear track images captured by a confocal microscope after the friction and wear experiments. The overall wear track is approximately 1 mm long, with different colors indicating variations in height. In images (a) to (e), it is clear that the ends of the wear track exhibit a build-up of material, particularly noticeable as raised areas at the edges of the track, represented by the yellow and red regions. This phenomenon is primarily due to the shearing and compressive effects of the ball on the surface of the overlay layer during the friction process, causing material from the coating to accumulate around the wear track. At a laser power of 2200 W, the coating demonstrates better friction performance, resulting in a relatively shallow wear track. This indicates that the high-power laser overlay treatment may help enhance the wear resistance of the material, reducing the depth of wear and thereby extending its service life. These observations provide important insights for further optimizing the tribological performance of the coating materials.

## 4. Conclusions

Using laser cladding technology, the effects of different laser powers on the microstructure (phase and microstructure) and mechanical properties (microhardness and wear resistance) of Stellite 12 cobalt-based alloy coatings were investigated. The main conclusions are as follows:

1. Stellite 12 coatings were prepared on the surface of H13 steel using different laser powers. Observations showed that at lower powers (1800–2000 W), there were unmolten powders on the surface and noticeable crack defects internally. At higher powers, a larger heat-affected zone appeared on the substrate surface, and internal crack defects were also present. At a laser power of 2200 W, the surface formation quality of the cladding layer was good, with no significant internal defects observed.
2. Examination of the internal microstructure revealed that at lower powers (1800–2000 W), there were many incomplete irregular grains, while at higher laser powers, the internal grains became coarser. At 2200 W, the internal grain arrangement was dense and well formed. Phase studies indicated that as the laser power increased, the diffusion of Fe into the coating led to a gradual increase in dilution rate. The cladding layer mainly consisted of γ-Co (face-centered cubic structure), M_7_C_3_, and M_23_C_6_ (where M represents Co, Cr, Mo, and W).
3. Microhardness testing indicated that increasing the laser power led to an overall decrease in coating hardness, and changes in the internal structure and chemical composition of the coating significantly affected hardness distribution. Friction and wear experiments showed that at a laser power of 2200 W, the coating exhibited good friction performance, with a friction coefficient of 0.57 and a wear volume of only 0.0162 mm^3^.

## Figures and Tables

**Table 1:** Composition of H13 steel and Stellite 12 powder.

Material	Mass Fraction/%								
	C	Cr	Si	W	Fe	Mo	Ni	Mn	Co
Stellite12	1.40	29.50	1.45	8.25	3.00	1.00	3.00	1.00	Bal.
H13	0.42	5	0.89	-	Bal.	1.27	0.16	0.3	-

**Table 2:** Process parameters for laser cladding.

Process Parameters	Parameter Data
Spot Shape at Intersection	square
Spot Size (mm)	3 × 3
Defocus Amount H (mm)	15
Powder Feed Rate Vr (r/min)	1.2
Laser Power P (W)	1800; 2000; 2200; 2400; 2600
Scanning Speed V_C_ (mm/min)	600
Overlap Rate (%)	40
Offset L (mm)	1.8
Shielding Gas Flow Rate L (min)	20
Waiting time (s)	0.4

## Data Availability

The original contributions presented in this study are included in the article. Further inquiries can be directed to the corresponding author.
